# Prognostic significance and therapeutic potential of guanosine triphosphate cyclohydrolase 1 in esophageal squamous cell carcinoma: clinical implications of ferroptosis and lipid peroxidation regulation

**DOI:** 10.3389/fonc.2024.1459940

**Published:** 2024-12-11

**Authors:** Masayoshi Sakano, Yoshinobu Tomita, Takumi Kanazawa, Sachiko Ishibashi, Masumi Ikeda, Haruna Oshita, Yuri Hananoi, Yuki Kato, Kurara Yamamoto, Asuka Furukawa, Mayumi Kinoshita, Shigeo Haruki, Masanori Tokunaga, Yusuke Kinugasa, Morito Kurata, Masanobu Kitagawa, Kenichi Ohashi, Kouhei Yamamoto

**Affiliations:** ^1^ Department of Gastrointestinal Surgery, Graduate School of Medical and Dental Sciences, Tokyo Medical and Dental University, Tokyo, Japan; ^2^ Department of Human Pathology, Graduate School of Medical and Dental Sciences, Tokyo Medical and Dental University, Tokyo, Japan; ^3^ Department of Clinical Laboratory Medicine, Faculty of Health Science Technology, Bunkyo Gakuin University, Tokyo, Japan; ^4^ Department of Comprehensive Pathology, Graduate School of Medical and Dental Sciences, Tokyo Medical and Dental University, Tokyo, Japan

**Keywords:** esophageal squamous cell carcinoma, guanosine triphosphate cyclohydrolase 1, ferroptosis, lipid peroxidation, prognosis, therapeutic strategy

## Abstract

**Background:**

Esophageal cancer, particularly esophageal squamous cell carcinoma (ESCC), is a leading cause of cancer-related death and has a poor prognosis. Despite the advancements in multidisciplinary therapies, resistance to conventional treatments warrants the development of novel therapeutic strategies. Ferroptosis, a form of cell death dependent on intracellular iron, has emerged as a potential mechanism for targeting cancer cells resistant to apoptosis. Guanosine triphosphate cyclohydrolase 1 (GCH1) has been identified as a novel antagonist of ferroptosis; however, its role in ESCC remains unclear. This study aimed to investigate the correlation between the expression and accumulation of the lipid peroxidation markers and regulators, including GCH1, in patients with ESCC and examined their prognostic significance. Furthermore, we investigated the relationship between lipid peroxidation regulators and cell death using an *in vitro* system to establish the basis for new therapeutic strategies.

**Methods:**

We retrospectively analyzed 312 patients with ESCC who underwent radical esophagectomy at the Tokyo Medical and Dental University. Immunohistochemistry was performed to evaluate the expression of lipid peroxidation markers (4-hydroxy-2-nonenal) and regulators (glutathione peroxidase 4 [GPX4], ferroptosis suppressor protein 1 [FSP1], and GCH1). The correlation between these markers, clinicopathological features, and overall survival was assessed. *In vitro* experiments were performed using KYSE-150 cells to investigate the effects of GCH1 knockdown and overexpression on cell proliferation, cisplatin-induced cell death, and ferroptosis.

**Results:**

Low GCH1 expression was significantly associated with a poor prognosis in patients with ESCC. GCH1 expression correlated with lymph node metastases, vessel invasion, and the pathological tumor stage. *In vitro*, GCH1-knockdown cells exhibited increased proliferation and resistance to cisplatin-induced cell death, whereas GCH1 overexpression reduced cell proliferation. Simultaneous inhibition of GPX4 and FSP1 induced mild cell death; however, GCH1 knockdown dramatically enhanced ferroptosis, suggesting a synergistic effect.

**Conclusion:**

GCH1 is a critical prognostic factor for ESCC and plays a significant role in the regulation of cell proliferation and ferroptosis. Targeting GCH1 in combination with GPX4 and FSP1 inhibitors may offer a novel therapeutic strategy for overcoming resistance in ESCC. Further studies are warranted to elucidate the involved molecular mechanisms and validate these findings *in vivo*.

## 1 Introduction

Esophageal cancer is the sixth leading cause of cancer-related deaths and the seventh most common malignancy worldwide (1); In 2018, 572,034 individuals were diagnosed with esophageal cancer and 508,585 people died from the disease worldwide (2). The two most common histological types of esophageal cancer are squamous cell carcinoma, which accounts for approximately 90% of the cases, and adenocarcinoma (3). Esophageal cancer has one of the poorest prognoses, with a 5-year survival rate of <30% (4). Multidisciplinary therapies, such as surgical resection, chemotherapy, radiation therapy, and chemoradiotherapy, have been developed for the treatment of esophageal cancers, including esophageal squamous cell carcinoma (ESCC) (5, 6). Although apoptosis-inducing agents, such as fluorouracil, platinum, and taxanes, remain the mainstay of drug therapy for the treatment of esophageal cancer, resistance has emerged as a considerable challenge, warranting the development of novel drug strategies for patients with ESCC (7–10).

In 2012, ferroptosis, a form of intracellular iron-dependent cell death, was identified, which is a distinct cell death mechanism, separate from apoptosis and necrosis (11). Ferroptosis plays a role in various organ injuries and degenerative diseases, and its pharmacological regulation promises the treatment of various cancers and ischemic organ damages (12). In particular, ferroptosis can provide effective cell death in refractory cancers (11). Therefore, it is a promising treatment strategy for poor-prognosis cancers.

In the last decade, a series of molecular mechanisms for the control of ferroptosis have been elucidated. In 2014, glutathione peroxidase 4 (GPX4) was reported as the main inhibitor of ferroptotic cell death (13). In 2019, ferroptosis suppressor protein 1 (FSP1), previously known as apoptosis-inducing factor mitochondria-related 2, was reported to function as an inhibitor of ferroptosis in a pathway independent of GPX4 and glutathione (14, 15). In 2020, guanosine triphosphate cyclohydrolase 1 (GCH1) was reported as the third novel antagonist of ferroptosis (16). In addition to these lipid peroxidation regulators, 4-hydroxy-2-nonenal (4-HNE) is known as an important lipid peroxidation marker (17). Various studies have reported on the expression and accumulation of the lipid peroxidation regulators and markers in cancer tissues; their expression varies based on the carcinoma type (18–23). Although studies have reported on the association between lipid peroxidation markers and the prognosis in esophageal cancer (24), the association with the accumulation of lipid peroxidation markers and GCH1 has not been investigated.

In this study, we investigated the correlation between the expression and accumulation of the lipid peroxidation markers and regulators, including GCH1, in patients with ESCC and examined their prognostic significance. Furthermore, we investigated the relationship between lipid peroxidation regulators and cell death using an *in vitro* system to establish the basis for new therapeutic strategies.

## 2 Materials and methods

### 2.1 Patients and pathological samples

This study included 312 patients with ESCC who underwent curative esophagectomy at Tokyo Medical and Dental University between January 1, 2007 and December 31, 2018. Patients were selected based on the following inclusion criteria: 1. Indication and performance of curative esophagectomy; 2. confirmed diagnosis of ESCC based on postoperative pathological diagnosis; and 3. complete clinical, operative, and pathological data. We retrospectively searched the databases and medical records of 312 patients and collected their clinical, surgical, and pathological data. The pathological tumor stage (pStage) was grouped according to the eighth edition of the tumor, nodes, and metastases classification developed by the Union for International Cancer Control (25). Informed consent was obtained whenever possible, and in cases where it was not possible to obtain consent, the content of this study was disclosed on the Tokyo Medical and Dental University Hospital website using an opt-out system as a substitute for informed consent. The study protocol was approved by the Tokyo Medical and Dental University Faculty of Medicine Ethics Review Committee (Number: M2000-1706).

### 2.2 Immunohistochemistry

Immunohistochemistry for 4-HNE, GPX4, FSP1, and GCH1 was performed using the avidin-biotin complex (ABC) or intercalating antibody-enhanced polymer methods. ESCC tissues fixed in neutral buffered formalin solution and embedded in paraffin were cut into 4 µm-thick sections and deparaffinized with xylene and ethanol series. These sections were subjected to antigen retrieval by heat, blocking of endogenous peroxidase with 3% hydrogen peroxide, and blocking with normal serum. The sections were subsequently incubated overnight at 4°C with primary antibodies against 4-HNE, GPX4, FSP1, and GCH1. Antibodies against GPX4, FSP1, and GCH1 were detected using an ABC kit (VECTASTAIN ABC kit; Vector Laboratories, Newark, CA, USA), and those against 4-HNE were detected using the Novolink Polymer Detection System (Leica, Wetzlar, Germany). Color development was performed using diaminobenzidine (Nichirei Bioscience, Tokyo, Japan). **Table 1** lists details of the staining conditions. The dilution rate for each antibody is shown in the concentration section of **Table 1**. Regarding the method of enhancing detection, we chose the polymer method for staining with anti-4-HNE antibody because the background staining was relatively strong in the ABC method.

**Table 1:** Primary antibody and conditions for immunostaining.

Antibody				
	4-HNE	GPX4	FSP1	GCH1
Source	JaICA	Abcam	ATRAS ANTIBODIES	SIGMA
Clone	HNEJ-2	ERNCIR144	HPA042309	HPA028612
Host	Mouse	Rabbit	Rabbit	Rabbit
Clonality	Monoclonal	Polyclonal	Monoclonal	Polyclonal
Lot number	011 MHN-100P	1000287-2	000018848	B114988
IHC condition				
Antigen retrieval	MW, 97°C, 20 min	MW, 97°C, 20 min	MW, 97°C, 20 min	MW, 97°C, 20 min
Buffer	pH 6.0 citrate buffer	pH 6.0 citrate buffer	pH 6.0 citrate buffer	pH 9.0 HISTOFINE
Concentration	× 200	× 500	× 250	× 500
Method	iAEP	ABC	ABC	ABC

4-HNE, 4-hydroxy-2-nonenal; GPX4, glutathione peroxidase 4; FSP1, ferroptosis suppressor protein 1; GCH1, guanosine triphosphate cyclohydrolase 1; IHC, immunohistochemistry; iAEP, intercalating antibody-enhanced polymer; ABC, avidin-biotin complex.

### 2.3 Evaluation of the staining intensity and correlation analysis

The cytoplasmic expression levels of 4-HNE, GPX4, FSP1, and GCH1 were evaluated, and the histochemical score (H-score) was calculated using the staining intensity and percentage of positive cells. Staining intensity was scored as 0, negative; 1, weak; and 2, strong. For 4-HNE, the nuclear expression levels were also evaluated, and H-scores were calculated. For the cytoplasmic expression, the staining intensity was scored as 0, negative; 1, weak; and 2, strong. 4-HNE(C) and 4-HNE(N) indicate cytoplasmic and nuclear expression, respectively. Specimens were divided into two groups based on the median H-score: high- and low-expression groups for specimens with H-scores above and below the median, respectively.

### 2.4 Clinicopathological analysis

For the clinicopathological analysis, we evaluated the following parameters: age, sex, smoking habit, alcohol consumption, differentiation, lymph node metastases, lymphovascular invasion, vessel invasion, and ESCC pStage. We analyzed the correlation between the clinical parameters; GPX4, FSP1, and GCH1 expression; 4-HNE accumulation; and the relationship between the prognosis and accumulation of the lipid peroxidation regulators and markers. Based on these results, we identified the independent prognostic factors using univariate and multivariate analyses.

### 2.5 Cell lines and tissue culture conditions

KYSE-150 cells, which were used for *in vitro* analysis, were obtained from the Japanese Collection of Research Bioresources (Osaka, Japan). The cells were cultured in RPMI-1640 medium containing L-glutamine and phenol red (Wako Pure Chemical Industries, Ltd., Osaka, Japan), 10% fetal bovine serum, and 1% penicillin-streptomycin. The cells were passaged at a ratio of 1:10 every 2–3 days.

### 2.6 Real-time quantitative polymerase chain reaction

Each cell was collected at a concentration of 1 × 10^6 cells/mL. RNA was extracted from freshly frozen ESCC samples using the RNeasy Mini Kit (QIAGEN, Hilden, Germany). The absorbance of 2 µL of the extracted RNA was measured using a DeNovix^®^ (Scrum Inc., cat.no DS-11+, Tokyo, Japan) to calculate the RNA concentration, and it was confirmed that the RNA quality was sufficient. Based on the calculated concentration, 200 ng of RNA was used for the reverse-transcription (RT) reaction. The RT reaction was performed using ReverTra Ace qPCR Master Mix (TOYOBO, Osaka, Japan) and TaKaRa PCR Thermal Cycler Dice^®^ Touch (Takara Bio Inc., Shiga, Japan) with the following conditions: 37°C for 15 min, 50°C for 5 min, and 98°C for 5 min. Using 1 µL of the generated cDNA as a template, a real-time qPCR was performed with PowerUp™ SYBR™ Green Master Mix (Thermo Fisher Scientific, Waltham, MA, USA) and the QuantStudio 3 Real-Time PCR System (Thermo Fisher Scientific). The qPCR protocol involved an initial step at 50°C for 2 min, followed by 95°C for 2 min, and then 40 cycles of 95°C for 15 s and 60°C for 1 min. Following this, a reaction at 95°C for 15 s and 60°C for 1 min was performed to confirm the occurrence of PCR reactions specific to the primers and target genes, and the melting curve was confirmed. Primers for GCH1 and β-actin were used with the following sequences: GCH1, 5′- CGGCCATGCAGTTCTTCACC-3′ (forward) and 5′- TGTCCTTCACAATCACCATCTCAT-3′ (reverse); β-actin, 5′- CACAGAGCCTCGCCTTTGCC-3′ (forward) and 5′-ACATGCCGGGAGCCGTTGTC-3′ (reverse) (Thermo Fisher Scientific).

### 2.7 Western blotting

The collected cells were dissolved by sonication in sodium dodecyl sulfate sample buffer containing Tris-HCl, glycerol, 10% sodium dodecyl sulfate, 2-mercaptoethanol, and bromophenol blue, and the cell lysates were incubated at 95°C for 10 min. Proteins were subjected to sodium dodecyl sulfate-polyacrylamide gel electrophoresis at 30 mA for 40 min, and the separated proteins were electroblotted onto polyvinylidene fluoride membranes at 120 V for 1 h. We blocked the membrane with Bullet Blocking one (NACALAI TESQUE, Inc., Kyoto, Japan) and incubated it with the primary antibody (1:1000 dilution) against GCH1 (Sigma-Aldrich, Burlington, MA, USA) at 4°C overnight. We washed the membrane using phosphatase-buffered saline with Tween 20 (Tris-buffered saline with Tween 20) two times for 10 min and incubated it with a secondary antibody (Cytiva, Tokyo, Japan) for 1 h at 20–25°C. The cells were then washed with Tris-buffered saline with Tween-20. We developed the blot using an enhanced chemiluminescence substrate (BIO-Rad Laboratories, Hercules, CA, USA) and ChemiDoc Touch MP (BIO-Rad Laboratories).

### 2.8 Establishment of GCH1-knockdown KYSE-150 cell line by lentiviral shRNA silencing of GCH1

We selected the KYSE150 cell line, which is positive for the *PT53* mutation, the most common gene mutation in esophageal cancer. GCH1-knockdown KYSE-150 cells were generated using shRNA lentiviral vectors. The shRNA lentiviral vectors used for *GCH1* were pLV[shRNA]-Puro-U6-Scramble_shRNA#1 and pLV[shRNA]-Puro-U6-hGCH1 (VectorBuilder, Kanagawa, JAPAN). The shRNA lentiviral vectors were transformed into *Escherichia coli* DH5α competent cells (Competent Quick DH5a [TOYOBO]) and the plasmid DNA was collected.

The pLV[shRNA]-Puro-U6-Scramble_shRNA#1 and pLV[shRNA]-Puro-U6-hGCH1 vectors were transfected into the HEK293T cells and 24 h later, the supernatant was replaced with the medium; 48 h following transfection, the supernatant was collected and used to infect the KYSE-150 cells by spin infection (4680 rpm, 30 min). Selection was performed with 1 µg/mL puromycin (Invitrogen, Waltham, MA, USA) 48 h after infection, and bulk cells were used for subsequent experiments. Knockdown efficiency in the KYSE-150 cells was determined by quantitative RT PCR (qRT-PCR) (forward primer: 5′-CGGCCATGCAGTTCTTCACC -3,’ reverse primer: 5′-TGTCCTTCACAATCACCATCTCAT -3′) at the mRNA level and protein level by western blotting.

### 2.9 Establishment of GCH1-overexpressing KYSE-150 cell line using recombinant lentiviruses and lentiviral transduction

GCH1-overexpressing KYSE-150 cells were established using lentiviral vectors overexpressing *GCH1.* The lentiviral vector used to overexpress *GCH1* was pLV[Exp]-Puro-EF1A-hGCH1[NM_000161.3] (VectorBuilder). pLV[Exp]-Puro-EF1A-ORF_Stuffer (VectorBuilder) was used as a control vector. These overexpressing lentiviral vectors were transformed into *E. coli* DH5α competent cells (Competent Quick DH5a [TOYOBO]) and the plasmid DNA was collected.

The pLV[Exp]-Puro-EF1A-ORF_Stuffer and pLV[Exp]- Puro-EF1A-hGCH1[NM_000161.3] vectors were transfected into the HEK293T cells and 24 h later, the supernatant was replaced with the medium. Forty-eight hours after transfection, the supernatant was collected and used to infect the KYSE-150 cells by spin infection (4680 rpm, 30 min). Selection was performed with 1 µg/mL puromycin (Invitrogen) 48 h after infection, and bulk cells were used for subsequent experiments. Knockdown efficiency in the KYSE-150 cells was determined by qRT-PCR (forward primer: 5′-CGGCCATGCAGTTCTTCACC -3,’ reverse primer: 5′-TGTCCTTCACAATCACCATCTCAT -3′) at the mRNA level and protein level by western blotting.

### 2.10 Evaluation of the proliferation of GCH1-knockdown and GCH1-overexpressing KYSE-150 cells

The proliferation of GCH1-knockdown and GCH1-overexpressing KYSE-150 cells was evaluated as follows: GCH1-knockdown KYSE-150 cells were seeded in 24-well culture plates at a density of 3 × 10^4^ cells/mL and GCH1-overexpressing KYSE-150 cells were seeded in 24-well culture plates at a density of 4 × 10^4^ cells/mL. After incubation at 37°C, the cells were harvested at 24, 48, and 72 h, and the cell proliferation capacity was confirmed by counting the number of cells with a Countess II FL automated cell counter (Thermo Fisher Scientific). GCH1-knockdown cells have relatively stronger cell proliferation than GCH1-overexpression cells. Therefore, in an experiment using the same number of cells, the cells reached confluence, and cell death was induced. To prevent this, the number of cells at 0 h was relatively reduced in the GCH1-knockdown cells used in this experiment. Each experiment was repeated twice.

### 2.11 Analyses of the cell viability upon treatment of the GCH1-knockdown and GCH1-overexpressing KYSE-150 cells with Cisplatin

The percentage of dead cells in the GCH1-knockdown and GCH1-overexpressing KYSE-150 cells upon cisplatin treatment was evaluated as follows: GCH1-knockdown and GCH1-overexpressing KYSE-150 cells were seeded in 6-well culture plates at a density of 2 × 10^5^ cells/2 mL at 37°C. After incubation for 24 h, cisplatin was added at 0 µM and 20 µM for the GCH1-knockdown KYSE-150 cells and 0 µM and 15 µM for the GCH1-overexpressing KYSE-150 cells. After incubation for 48 h, the cells were collected, the live and dead cells were counted using a Countess II FL automated cell counter, and the percentage of dead cells was calculated. Each experiment was repeated twice.

### 2.12 Cell death analysis following treatment with GPX4 and FSP1 inhibitors

The cells were treated with two combinations of inhibitors to evaluate the synergistic effects of the reagent combinations on dead cells: a GPX4 inhibitor (RSL3; MedChemExpress, Tokyo, Japan) and an FSP1 inhibitor (iFSP1; R&D Systems, Minneapolis, MN, USA), and a GPX4 inhibitor (ML210; Sigma-Aldrich Japan, Tokyo, Japan) and an FSP1 inhibitor (iFSP1; R&D Systems). GCH1-knockdown KYSE-150 cells were seeded in 96-well culture plates at a density of 1 × 10^4^ cells/100 µL and incubated at 37°C for 24 h, then treated with RSL3 at final concentrations of 0, 50, 100, 200 µM and iFSP1 at 0, 5, 10, 20 µM and ML210 at 0; ML210 was administered at concentrations of 0, 5, 10, and 20 µM and iFSP1 at concentrations of 0, 5, 10, and 20 µM. The Cytotoxicity LDH Assay Kit-WST (DOJINDO LABORATORIES, Kumamoto, Japan) was used to measure the cytotoxicity 24 h after administration. For Cytotoxicity LDH Assay Kit-WST, 100 µL of working solution was added 24 h after administration of the inhibitor; after shading from light for 30 min at room temperature, 50 µL of stop solution was added to stop the reaction. Cell damage was assessed by measuring the absorbance at 490 nm using a microplate reader ELx808 (Agilent, Tokyo, Japan). The cytotoxicity control was defined as low (no drug added) and high (treated with lysis buffer) control, and the percentage of cell death was calculated as 0% and 100%, respectively. Each experiment was repeated twice.

### 2.13 Analyses of the cell death properties using a cell death inhibitory reagent

The effects of cell death inhibitors on the cell death, caused by two different ways, among GCH1-knockdown KYSE-150 cells were investigated. The following combinations were used: RSL3 and iFSP1, and ML210 and iFSP1; 200 µM RSL3, 20 µM iFSP1, 400 µM ML210, and 20 µM iFSP1 were treated simultaneously with the cell death inhibitors. The cell death inhibitors included 2 µM ferrostatin-1 (ferroptosis inhibitor; Sigma-Aldrich, Burlington, MA, USA), 250 µM Z-VAD-FMK (apoptosis inhibitor; Peptide Institute, Osaka, Japan), and 100 µM necrostatin-1 (necrosis inhibitor; Adipogen Life Science, San Diego, CA, USA). GCH1-knockdown KYSE-150 cells were seeded in 96-well culture plates at a density of 1×10^4^ cells/100 µL, incubated at 37°C for 24 h, and then treated with the above-mentioned cell death inhibitors. Twenty-four hours after treatment, cytotoxicity was measured using the Cytotoxicity LDH Assay Kit-WST, and the percentage of cell death was calculated. Measurements and calculations were performed as described in the aforementioned cell death analysis following treatment with the GPX4 and FSP1 inhibitors. Each experiment was repeated twice.

### 2.14 Statistical analysis

The association between the two groups was evaluated using Fisher’s exact test. Overall survival (OS) was defined as the time from the date of diagnosis to the date of the last follow-up or death. The Kaplan–Meier survival curve was used to evaluate the OS, and the log-rank test was used to evaluate the statistical differences between the two groups. Univariate and multivariate analyses were performed using Cox proportional hazards regression models to evaluate independent factors of OS. Student’s t-test was used to evaluate the significant differences in all the *in vitro* analyses; *p* < 0.05 was considered statistically significant. All the statistical analyses were performed using JMP version 17 (SAS Institute, Inc. Cary, NC.) and GraphPad Prism version 9.1.2 (GraphPad Software, San Diego, CA, USA).

## 3 Results

### 3.1 Measurement of the H-scores of 4-HNE, GPX4, FSP1, and GCH1

**Figure 1:**
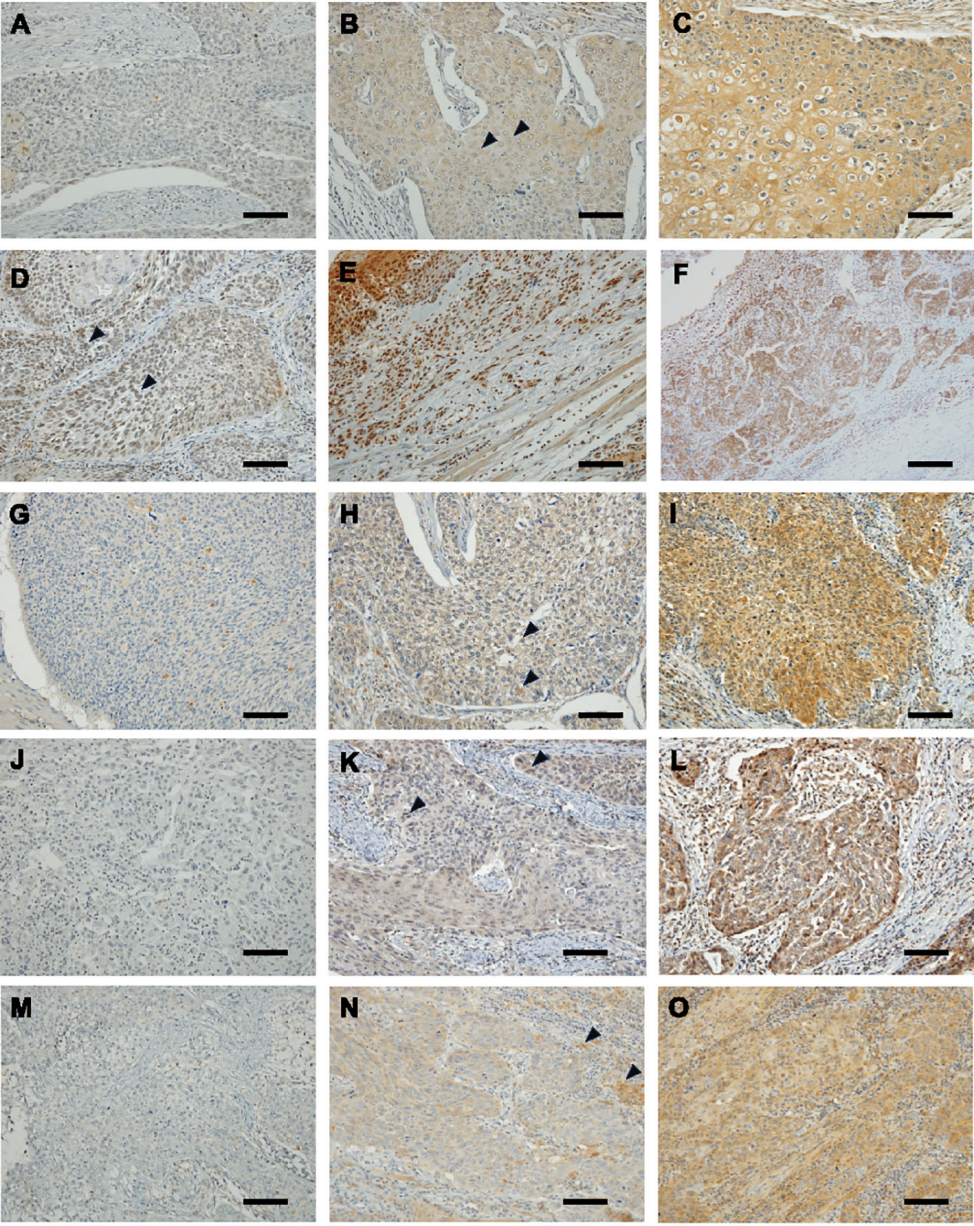
Immunohistochemical staining of esophageal squamous cell cancer (ESCC) for 4-hydroxy-2-nonenal (4-HNE), glutathione peroxidase 4 (GPX4), ferroptosis suppressor protein 1 (FSP1) and guanosine triphosphate cyclohydrolase 1 (GCH1). Images are shown at × 200 magnification; scale bar = 100 μm. **(A)**: Representative case of negative 4-HNE accumulation in both the cytoplasm and nucleus. **(B)**: Representative case of weak accumulating 4-HNE in the cytoplasm. Black arrowheads indicate weak positivity for 4-HNE in the cytoplasm. **(C)**: Representative case of strong accumulating 4-HNE in the cytoplasm. **(D)**: Representative case of weak accumulating 4-HNE in the nucleus lack arrowheads indicate weak positivity for 4-HNE in the nucleus. **(E)**: Representative case of strong accumulating 4-HNE in the nucleus. **(F)**: Representative case of strong accumulating 4-HNE both in the cytoplasm and nucleus. **(G)**: Representative case of negative expressing GPX4 ESCC. **(H**): Representative case of weak expressing GPX4 ESCC. Black arrowheads indicate weak positivity for GPX4. **(I)**: Representative case of strong expressing GPX4 ESCC. **(J)**: Representative case of negative expressing FSP1 ESCC. **(K)**: Representative case of weak expressing FSP1 ESCC. Black arrowheads indicate weak positivity for FSP1. **(L)**: representative case of strong expressing FSP1 ESCC. **(M)**: Representative case of negative expressing GCH1 ESCC. **(N)**: Representative case of weak expressing GCH1 ESCC. Black arrowheads indicate weak positivity for GCH1. **(O)**: Representative case of strong expressing GCH1 ESCC.

Immunostaining results for 4-HNE, GPX4, FSP1, and GCH1 are shown in **Figure 1**. The H-score was used to objectively evaluate the staining intensity of the specimens. Based on the median H-scores of 4-HNE, GPX4, FSP1, and GCH1, we divided the specimens into two groups: high-and a low-expression groups for specimens above and below the median, respectively. Regarding the 4-HNE immunostaining results, we evaluated the H-scores of the cytoplasm and nucleus separately. Based on the results of the 4-HNE(C) immunostaining, 177 and 135 patients were divided into the high- and low-expression groups, respectively. The median H-score for 4-HNE(C) was 100. Based on the results of the 4-HNE(N) immunostaining, 159 and 153 patients were divided into the high- and low-expression groups, respectively. The median H-score for 4-HNE(N) was 40. Finally, 117 patients had H-scores above the median of 4-HNE(C) and 4-HNE(N), and 195 patients had H-scores below the median. Based on the results of GPX4 immunostaining, 147 and 165 patients were divided into the high- and low-expression groups, respectively. The median H-scores for GPX4 were 140. Similarly, according to the FSP1 immunostaining results, 163 and 149 patients were divided into high-and low-expression groups, respectively. The median H-score of the FSP1 group was 30. For GCH1, we divided 165 patients each into high- and low-expression groups. The median H-score for GCH1 was 65.

### 3.2 Evaluation of the correlation between the accumulation of 4-HNE and lipid peroxidation regulators in ESCC

Since 4-HNE, a lipid peroxidation marker, is thought to be inversely correlated with the negative regulators of lipid peroxidation, we investigated the relationship between the accumulation of 4-HNE and lipid peroxidation regulators. 4-HNE accumulation was evaluated by separating 4-HNE(C) from 4-HNE(N). Patients were also divided into two groups: those with H-scores above the median of both 4-HNE(C) and 4-HNE(N), and others. Contrary to expectations, a positive relationship was observed between 4-HNE(C) and GCH1 accumulation and between 4-HNE(N) and FSP1 or GCH1 accumulation (**Tables 2**, **3**). In addition, a positive relationship was observed between the above two groups and FSP1 and GCH1 (**Table 4**).

**Table 2:** Correlation between 4-HNE(C) accumulation and the lipid peroxidation regulator expression levels.

GPX4					
		Low	High	r^a^	*p*-value
**4-HNE(C)**	**Low**	69	66	0.202	0.256
	**High**	79	98		
FSP1					
		Low	High	r^a^	*p*-value
**4-HNE(C)**	**Low**	70	65	0.242	0.062
	**High**	73	104		
GCH1					
		Low	High	r^a^	*p*-value
**4-HNE(C)**	**Low**	77	58	0.218	**0.029**
	**High**	79	98		

a r: correlation coefficient.

4-HNE, 4-hydroxy-2-nonenal; GPX4, glutathione peroxidase 4; FSP1, ferroptosis suppressor protein 1; (C), cytoplasmic expression; GCH1, guanosine triphosphate cyclohydrolase 1.The meaning of the bold text and values indicates that there is a significant difference.

**Table 3:** Correlation between 4-HNE(N) accumulation and lipid peroxidation regulator expression levels.

GPX4					
		Low	High	r^a^	*p*-value
**4-HNE(N)**	**Low**	79	74	0.154	0.145
	**High**	69	90		
FSP1					
		Low	High	r^a^	*p*-value
**4-HNE(N)**	**Low**	90	63	0.417	**<0.001**
	**High**	53	106		
GCH1					
		Low	High	r^a^	*p*-value
**4-HNE(N)**	**Low**	96	57	0.449	**<0.001**
	**High**	60	99		

a r: correlation coefficient.

4-HNE: 4-hydroxy-2-nonenal, GPX4, glutathione peroxidase 4; FSP1, ferroptosis suppressor protein 1; GCH1, guanosine triphosphate cyclohydrolase 1; (N), nuclear expression.The meaning of the bold text and values indicates that there is a significant difference.

**Table 4:** Correlation between 4-HNE(C) and 4-HNE(N) accumulation and the expression of lipid peroxidation regulators.

GPX4					
		Low	High	r^a^	*p*-value
**4-HNE(C) and 4-HNE(N)**	**Others**	100	95	0.099	0.079
	**High**	48	69		
FSP1					
		Low	High	r^a^	*p*-value
**4-HNE(C) and 4-HNE(N)**	**Others**	109	86	0.261	**<0.001**
	**High**	34	83		
GCH1					
		Low	High	r^a^	*p*-value
**4-HNE(C) and 4-HNE(N)**	**Others**	118	77	0.271	**<0.001**
	**High**	38	79		

a r: correlation coefficient.

4-HNE, 4-hydroxy-2-nonenal; GPX4, glutathione peroxidase 4; FSP1, ferroptosis suppressor protein 1; GCH1, guanosine triphosphate cyclohydrolase 1; (C), cytoplasmic expression; (N), nuclear expression.The meaning of the bold text and values indicates that there is a significant difference.

### 3.3 Correlation between the clinicopathological features and accumulation of 4-HNE and lipid peroxidation regulators

We investigated the correlation between 4-HNE accumulation or the GPX4, FSP1, and GCH1, expression levels with the clinicopathological factors in ESCC. 4-HNE accumulation was evaluated by separating 4-HNE(C) and 4-HNE(N). Patients were also divided into two groups: those with H-scores above the median of both 4-HNE(C) and 4-HNE(N) and others. We found that 4-HNE(C) accumulation significantly correlated with vessel invasion (*p* = 0.017), and 4-HNE(N) accumulation significantly correlated with lymph node metastases (*p* = 0.033), vessel invasion (*p* < 0.001), and pStage (*p* = 0.024). These two groups significantly correlated with lymph node metastases (*p* = 0.037), vessel invasion (*p* < 0.001), and pStage (*p* = 0.032). GPX4 expression significantly correlated with differentiation (*p* = 0.001), and FSP1 expression significantly correlated with age (*p* = 0.038) and vessel invasion (*p* = 0.005). GCH1 expression significantly correlated with lymph node metastases (*p* < 0.001), vessel invasion (*p* < 0.001), and pStage (*p* < 0.001).

### 3.4 Prognostic analyses based on the 4-HNE, GPX4, FSP1, and GCH1 expression

**Figure 2:**
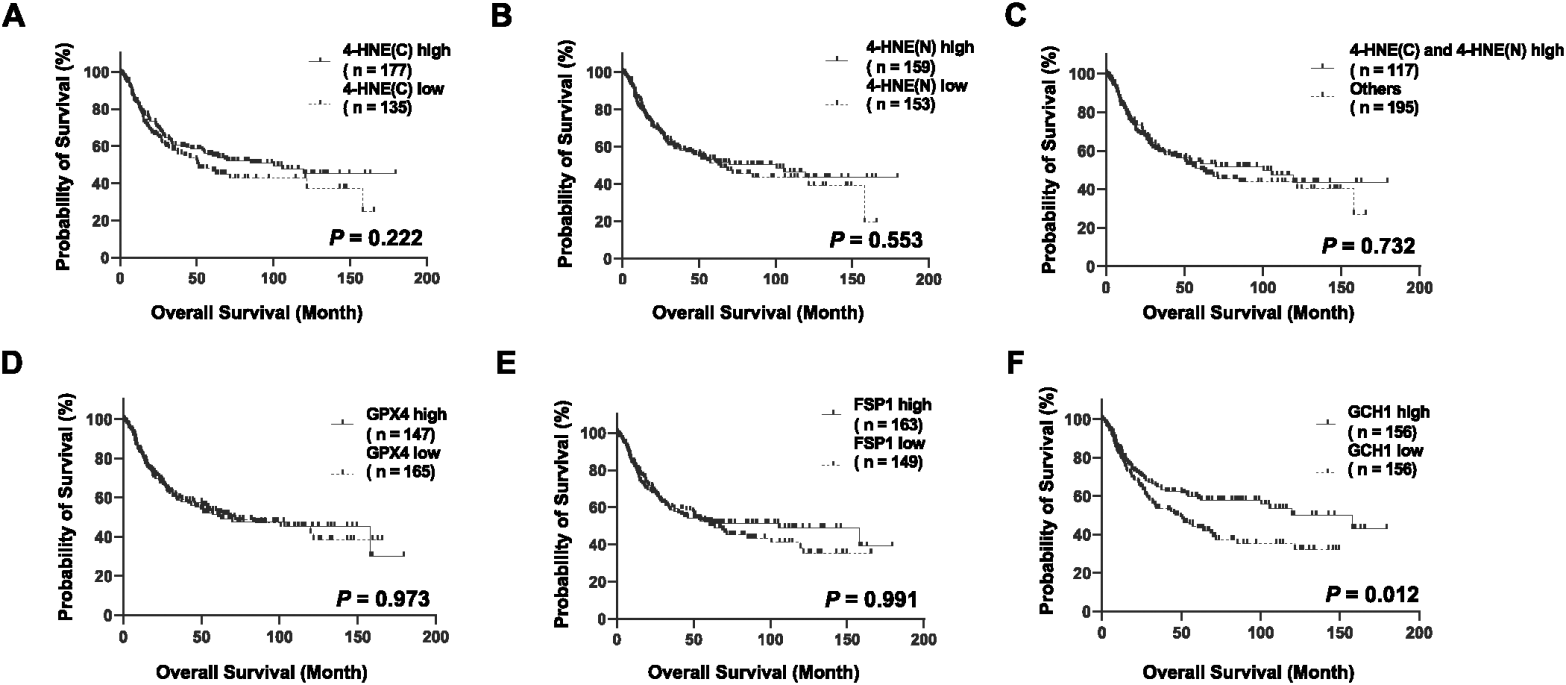
Overall survival (OS) analysis of esophageal squamous cell cancer (ESCC) using classification based on the histochemical score of 4-hydroxy-2-nonenal (4-HNE), glutathione peroxidase 4 (GPX4), ferroptosis suppressor protein 1 (FSP1), and guanosine triphosphate cyclohydrolase 1 (GCH1). Specimens are divided into two groups based on the median H-score: high-expression group for specimens with H-scores above the median and low-expression group for specimens below the median. Cytoplasm is shown as C, nucleus as N. **(A)**: Classification based on the accumulation of 4-HNE in the cytoplasm, showing no association with prognosis between the 4-HNE(C) high and low groups (*p* = 0.222). **(B)**: Classification based on the accumulation of 4-HNE in the nucleus, showing no association with prognosis between the 4-HNE(C) high and low groups (*p* = 0.553). **(C)**: In the high-expression intensity group, both 4-HNE(C) and 4-HNE(N) are not associated with prognosis (*p* = 0.732). **(D)**: No association with prognosis is found between GPX4 high and GPX4 low groups (*p* = 0.973). **(E)**: No association with prognosis is found between FSP1 high and FSP1 low groups (*p* = 0.991). **(F**): Patients with low GCH1 expression have a significantly poorer prognosis than those with high GCH1 expression (*p* = 0.012).

We stratified the patients according to the H-scores of 4-HNE, GPX4, FSP1, and GCH1, and analyzed the correlation between their accumulation or expression levels and OS. The median follow-up time was 32.1 months (range: 0.4–179.4 months). Kaplan–Meier analysis revealed that the expression levels of 4-HNE(C), 4-HNE(N), GPX4, and FSP1 were not associated with the 5-year OS. Moreover, the high-expression intensity groups, 4-HNE(C) and 4-HNE(N), were not associated with the 5-year OS (**Figures 2A–E**). In contrast, patients with low GCH1 expression had a significantly poorer prognosis than those with high GCH1 expression (**Figure 2F**, *p* = 0.012).

### 3.5 Correlation between the clinicopathological features in patients in the GCH1 high-expression group

We investigated the correlation between 4-HNE accumulation or the expression intensity of GPX4, FSP1, and GCH1 and the clinicopathological factors (**Supplementary Tables 1-5**). We found that high expression intensity of GCH1 significantly correlated with lymph node metastases, vessel invasion, and pStage (**Table 5**). We also investigated the correlation between the OS and 15 parameters, namely age, sex, smoking, alcohol consumption, differentiation, lymph node metastases, lymphovascular invasion, vessel invasion, pStage, 4-HNE accumulation, high expression intensity of both 4-HNE(C) and 4-HNE(N), and GPX4, FSP1, and GCH1 expression levels using the Cox univariate analysis. We found that a high expression intensity of GCH1 was associated with the OS in the univariate analysis (**Table 6**). In the multivariate analysis, sex (hazard ratio [HR], 2.163; *p* = 0.005) and pStage (HR, 0.394; *p* = 0.003) were independent predictors of poor prognosis (**Table 7**).

**Table 5:** Correlation between GCH1 accumulation and clinicopathological features.

Variable		GCH1		*p*-value
		Low	High	
**Age**	<69 y	73	80	0.427
	≥69 y	83	76	
**Sex**	Male	133	128	0.444
	Female	23	28	
**Smoking**	None	38	37	0.894
	Exist	118	119	
**Alcohol**	None	27	23	0.537
	Exist	129	133	
**Differentiation**	Well/moderate	106	98	0.341
	Poor/unknown	50	58	
**Lymph node metastases**	None	33	60	**<0.001**
	Exist	123	96	
**Lymphovascular invasion**	None	52	61	0.289
	Exist	104	95	
**Vessel invasion**	None	16	42	**<0.001**
	Exist	140	114	
**Pathological tumor stage**	I–II	46	82	**<0.001**
	III–IV	110	74	

GCH1, guanosine triphosphate cyclohydrolase 1.The meaning of the bold text and values indicates that there is a significant difference.

**Table 6:** Univariate analysis of the clinicopathological factors influencing the overall survival.

Variable	Category	No. of patients	*p*-value by log-rank test
**Age**	<69 y	153	0.601
	≥69 y	159	
**Sex**	Male	261	**0.021**
	Female	51	
**Smoking**	None	75	0.879
	Exist	237	
**Alcohol**	None	50	0.769
	Exist	262	
**Differentiation**	Well/moderate	204	0.406
	Poor/unknown	108	
**Lymph node metastases**	None	93	**<0.001**
	Exist	219	
**Lymphovascular invasion**	None	119	**<0.001**
	Exist	113	
**Vessel invasion**	None	254	**<0.001**
	Exist	58	
**Pathological tumor stage**	I–II	128	**<0.001**
	III–IV	184	
**4-HNE(C)**	Low	135	0.222
	High	177	
**4-HNE(N)**	Low	153	0.533
	High	159	
**4-HNE(C) and 4-HNE(N)**	Others	195	0.732
	High	117	
**GPX4**	Low	165	0.973
	High	147	
**FSP1**	Low	149	0.991
	High	163	
**GCH1**	Low	156	**0.012**
	High	156	

4-HNE, 4-hydroxy-2-nonenal; GPX4, glutathione peroxidase 4; FSP1, ferroptosis suppressor protein 1; GCH1, guanosine triphosphate cyclohydrolase 1; (C), cytoplasmic expression; (N), nuclear expression.The meaning of the bold text and values indicates that there is a significant difference.

**Table 7:** Multivariate analysis of the clinicopathological factors influencing the overall survival in all patients.

Variable	Category	No. of patients	HR^a^	95% Cl^b^	*p*-value by Cox proportional hazards	HR^a^	95% Cl^b^	*p*-value by Cox proportional hazards
**Age**	<69 y	153	0.955	0.670–1.362	0.800			
	≥69 y	159						
**Sex**	Male	261	2.163	1.249–3.743	**0.005**			
	Female	51						
**Smoking**	None	75	1.131	0.703–1.821	0.609			
	Exist	237						
**Alcohol**	None	50	1.155	0.698–1.910	0.502			
	Exist	262						
**Differentiation**	Well/moderate	204	0.885	0.619–1.264	0.502			
	Poor/unknown	108						
**Lymph node metastases**	None	93	1.235	0.640–2.381	0.528			
	Exist	219						
**Lymphovascular** **invasion**	None	119	0.696	0.449–1.080	0.106			
	Exist	113						
**Vessel invasion**	None	254	0.561	0.314–1.004	0.051			
	Exist	58						
**Pathological tumor stage**	I–II	128	0.394	0.213–0.728	**0.003**			
	III–IV	184						
**4-HNE(C)**	Low	135	1.079	0.756–1.540	0.672			
	High	177						
**4-HNE(N)**	Low	153	0.864	0.597–1.252	0.442			
	High	159						
**4-HNE(C) and 4-HNE(N)**	Others	195				1.118	0.796–1.569	0.518
	High	117						
**GPX4**	Low	165	0.953	0.669–1.357	0.791			
	High	147						
**FSP1**	Low	149	0.945	0.650–1.372	0.767			
	High	163						
**GCH1**	Low	156	0.859	0.597–1.236	0.414			
	High	156						

a HR: Hazard ratio.

b CI: Confidence interval.

4-HNE, 4-hydroxy-2-nonenal; GPX4, glutathione peroxidase 4; FSP1, ferroptosis suppressor protein 1; GCH1, guanosine triphosphate cyclohydrolase 1; (C), cytoplasmic expression; (N), nuclear expression.The meaning of the bold text and values indicates that there is a significant difference.

### 3.6 GCH1 expression influences the proliferation in ESCC

**Figure 3:**
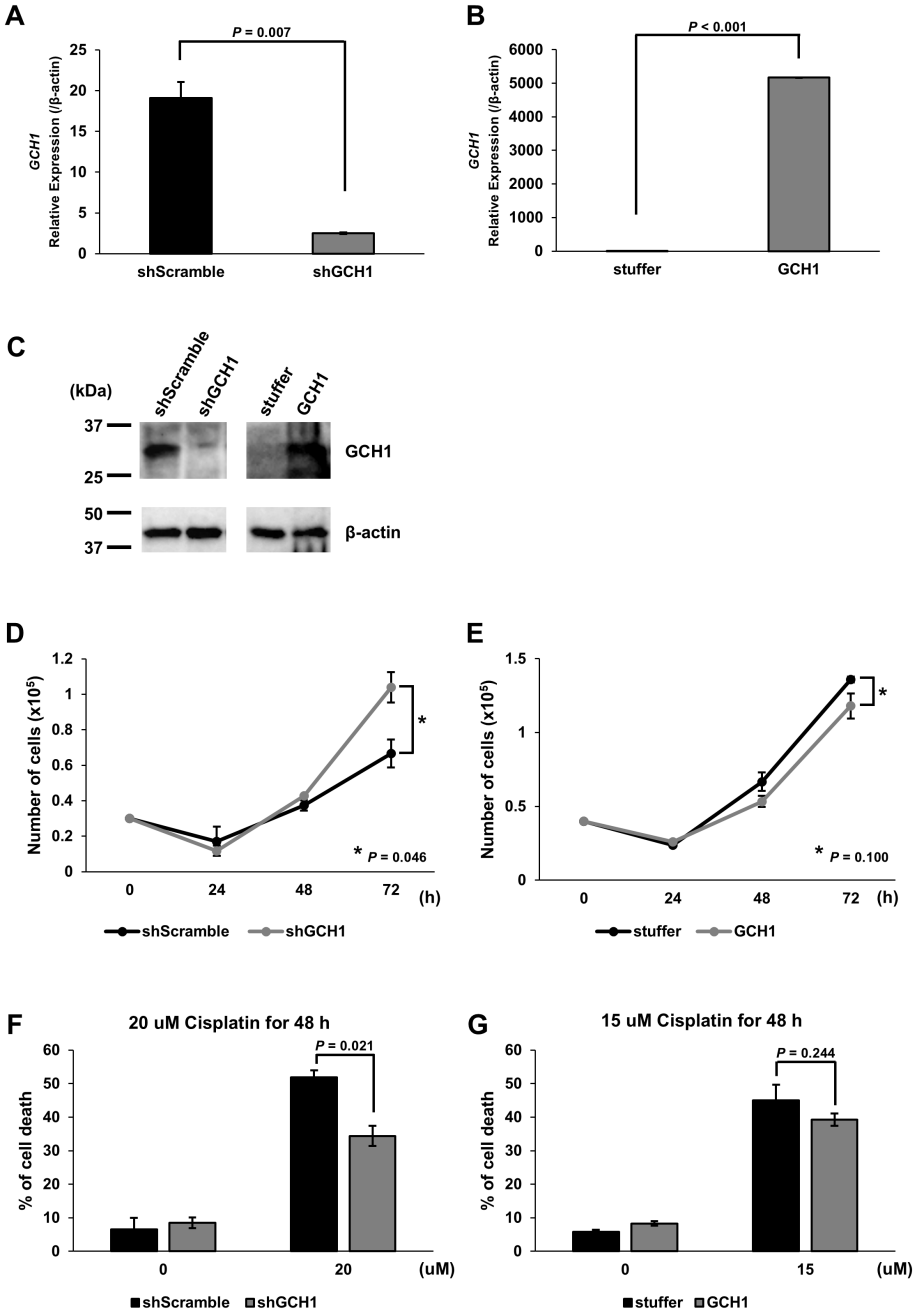
Expression of guanosine triphosphate cyclohydrolase 1 (GCH1) in esophageal cancer cell lines and its effects on cell proliferation and apoptosis stimulation. **(A)**: Quantitative reverse transcription polymerase chain reaction (qRT-PCR) showing GCH1 knockdown at the mRNA level in KYSE-150 cells (*p* = 0.007) **(B)**: qRT-PCR showing significant overexpression of GCH1 mRNA in KYSE-150 cells (*p* < 0.001). **(C)**: Knockdown and overexpression of GCH1 in KYSE-150 cells are detected at the protein level by western blotting. **(D)**: The number of GCH1-knockdown cells is significantly higher than that of control cells after 72 h (*p* = 0.046). **(E)**: The number of GCH1-overexpressing cells is lower than that of control cells after 72 h (*p* = 0.1). **(F)**: The cell death rate 48 h after cisplatin treatment is significantly suppressed in GCH1-knockdown cells compared to that in control cells (*p* = 0.021). **(G)**: Cell death rate is slightly lower than that of control cells 48 h after cisplatin treatment in GCH1-overexpressing cells (*p* = 0.244).

Based on the finding that the GCH1 low-expression group in ESCC correlates with poor prognosis, we hypothesized that GCH1 contributes to cancer malignancy. We considered that GCH1 is involved in enhancing cell proliferation and suppressing cell death and that GCH1 overexpression induces a decrease in tumor cell proliferation, whereas decreased GCH1 expression induces an increase in tumor cells. To test this hypothesis, we conducted experiments *in vitro*. GCH1-knockdown and GCH1-overexpressing KYSE-150 cells were established via transfection with a lentiviral vector. Decreased expression or overexpression of GCH1 in the cells was detected at the mRNA level by qRT-PCR and at the protein level by western blotting (**Figures 3A–C**). We measured the proliferation of GCH1-knockdown and GCH1-overexpressing cells. The number of GCH1-knockdown cells was significantly higher than that of the control cells (**Figure 3D**) 72 h later (*p* = 0.046). The number of GCH1-overexpressing cells was lower than that of the control cells after 72 h (**Figure 3E**) (*p* = 0.100).

### 3.7 GCH1-knockdown cell resistance to cisplatin-induced cell death

Using cisplatin, which is a chemotherapeutic agent that induces apoptosis and one of the most common treatments for esophageal cancer, cell experiments were performed to determine the presence of a difference in the response to the drug between cells overexpressing GCH1 and esophageal cancer cells in which GCH1 was knocked down. The survival of cisplatin-treated cells was determined. The cell death rate 48 h following cisplatin treatment was significantly suppressed in the GCH1-knockdown cells compared to that in the control cells (*p* = 0.021; **Figure 3F**). In the GCH1-overexpressing cells, the cell death rate was slightly lower than that in the control cells 48 h following cisplatin treatment (*p* = 0.244, **Figure 3G**); however, no significant difference was observed upon comparison with the control cells.

### 3.8 GCH1 inhibition enhances cell death by suppressing GPX4 and FSP1

**Figure 4:**
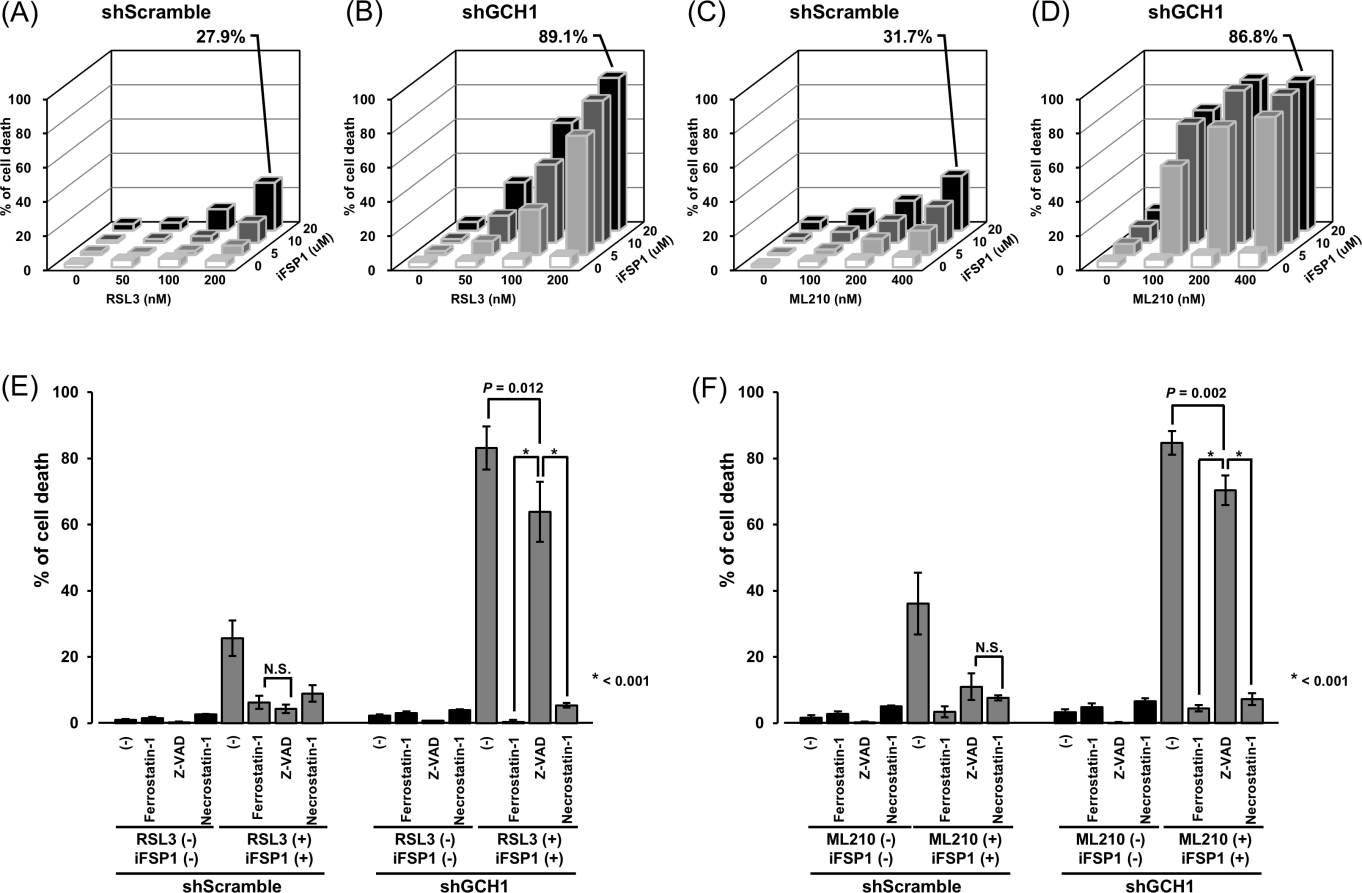
Suppression of guanosine triphosphate cyclohydrolase 1 (GCH1) enhances nonapoptotic cell death by inhibiting glutathione peroxidase 4 (GPX4) and ferroptosis suppressor protein 1 (FSP1). **(A)**: Cell death ratio after treatment with RSL3 (0, 50, 100, and 200 µM) and iFSP1 (0, 50, 10, and 20 µM) in control cells. **(B)**: Cell death ratio after treatment with RSL3 (0, 50, 100, and 200 µM) and iFSP1 (0, 50, 10, and 20 µM) in GCH1-knockdown cells. **(C)**: Cell death ratio after treatment with ML210 (0, 100, 200 and 400 µM) and iFSP1 (0, 50, 10, and 20 µM) in control cells. **(D)**: Cell death ratio after treatment with ML210 (0, 100, 200, and 400 µM) and iFSP1 (0, 50, 10, and 20 µM) in GCH1-knockdown cells. **(E, F)**: Cell death ratio in control cells and GCH1-knockdown cells treated with cell death inhibitors and 200 µM RSL3 and 20 µM iFSP1, and 400 µM RSL3 and 20 µM iFSP1. Cell death inhibitors included 2 µM ferrostatin-1, 250 µM Z-VAD-FMK and 100 µM necrostatin-1. Detailed cell death ratios and standard deviations for each experiment are given in **Supplementary Figure S1**. N.S.: nonsignificant.

As GCH1 depletion is reportedly involved in ferroptosis (16), we investigated the function of GCH1 in relation to ferroptosis in esophageal cancer. To examine the control and GCH1-knockdown cells, we performed cellular experiments using RSL3 and iFSP1, which inhibit GPX4 and FSP1, respectively. In the control cells, RSL3 or iFSP1 alone induced minimal cell death. When both drugs were combined, the percentage of cell death increased to 27.9% (**Figure 4A**). Similarly, in GCH1-knockdown cells, RSL3 or iFSP1 alone did not induce cell death, but the combination of both drugs induced cell death at a very high rate of 89.1% (**Figure 4B**). This effect was stronger than that observed in control cells.

To increase the level of evidence for this cellular experiment, we performed a cellular experiment using another GPX4 inhibitor, ML210. However, in the GCH1-knockdown cells, similar to that in the control cells, Ml210 or iFSP1 alone did not induce cell death to any significant extent; nevertheless, the percentage of cell death induced by the combination of both drugs increased to 31.7% (**Figure 4C**). In GCH-knockdown cells, similar to that in control cells, Ml210 or iFSP1 alone did not induce cell death to any significant degree, whereas RSL3 and iFSP1 together induced cell death at a rate as high as 86.8% (**Figure 4D**). Detailed cell death ratios and standard deviations for each experiment are given in **Supplementary Figure S1**.

To further elucidate the mechanism of the synergistic cell death induction effect in GCH1 control and GCH1-knockdown cells, experiments were conducted using various cell death inhibitors. The results demonstrated that in control cells exposed to RSL3 and iFSP1, cell death induction was inhibited by Z-VAD-FMK to the same extent as ferrostatin-1. In contrast, in the GCH1-knockdown cells, the inhibitory effect of Z-VAD on cell death induction was partially limited (control cell vs. GCH1-knockdown cell: 4.3% vs. 63.8%), and the inhibitory effect of Z-VAD-FMK was significantly reduced compared (*p* < 0.05) to that of ferrostatin-1 and necrostatin-1 (*p* < 0.001 and *p* < 0.001, respectively) (**Figure 4E**).

In the control cells exposed to ML210 and iFSP1, the induction of cell death was equally suppressed when Z-VAD and necrostatin-1 were used. However, in the GCH1-knockdown cells exposed to ML210 and iFSP1, cell death induction was suppressed by ferrostatin and necrostatin, similar to that in the combination of RLS3 and iFSP1; nevertheless, the suppression of cell death induction was limited by Z-VAD (control cell GCH1-knockdown cell: 10.9% vs. 70.4%), and the inhibitory effect of Z-VAD-FMK was significantly lower than that of ferrostatin-1 and necrostatin-1 (*p* < 0.001 and *p* < 0.001, respectively) (**Figure 4F**).

## 4 Discussion

Esophageal cancer has a poor prognosis, and many cases are resistant to chemotherapy, especially with apoptosis inhibitors. New antitumor strategies are being developed to improve the prognosis of patients with esophageal cancer (24).

In the present study, analysis of ESCC immunostaining and clinical data revealed that low GCH1 expression was a significantly poor prognostic factor, whereas no significant prognostic difference was observed for GPX4 and FSP1 expression. Previous reports have demonstrated that high FSP1 and GPX4 expression is a poor prognostic factor for esophageal cancer (24). However, in our study, no significant differences in the prognosis were observed. The antibodies used in the FSP1 and GPX4 studies were the same clones as those used in the present study; however, the staining intensity was evaluated by classifying the tumors as positive or negative based on the percentage of positively stained areas and then dividing them into two groups based on the median values. Therefore, the results may vary owing to differences in the evaluation methods.

Although the expression pattern of GCH1 in cancer tissues has not been well-documented, GCH1 is reportedly upregulated in breast and ovarian cancers (26), and that high GCH1 expression is reportedly associated with a shorter OS in triple-negative breast cancer (27). At the *in vitro* experimental level, GCH1 overexpression in glioblastoma cells reportedly increased cell proliferation *in vitro* and decreased survival in an intracranial glioblastoma mouse model, and GCH1 knockdown was associated with decreased CD44 expression, resulting in the suppression of glioblastoma cell proliferation and decreased self-renewal (28). Thus, GCH1 has been previously reported to yield a poor prognosis. Furthermore, the overexpression of GPX4 and FSP1 similarly has an advantage in terms of poor prognosis and cancer progression (29–32). In contrast, immunostaining of ESCC cells and analysis of clinical data revealed that low GCH1 expression was a significantly poor prognostic factor, and *in vitro* experimental results revealed that low GCH1 expression contributed to cell proliferation and resulted in resistance to cisplatin. Several possible reasons contribute to these results. First, quantitative changes in tetrahydrobiopterin (BH4) may affect GCH1 expression; GCH1 is known to be involved in BH4 synthesis, and GCH1 expression positively correlates with the amount of BH4 synthesized (15). However, a negative feedback mechanism reportedly exists in which GCH1 regulatory feedback protein binds to GCH1 and forms an inhibitory GCH1-GCH1 regulatory feedback protein complex, thereby reducing GCH1 activity when BH4 reaches a sufficient concentration (33). Thus, this negative mechanism and detection of proteins containing GCH1-GCH1 regulatory feedback protein complexes in the GCH1 high-expression group may have contributed to the good prognosis. Second, GCH1 may be downregulated because of its unknown function in cell death. It was recently reported that GCH1 promotes breast cancer and metastasis via epithelial-mesenchymal transition without BH4 (34). Such nonenzymatic functions of GCH1 may contribute to its favorable prognosis in esophageal cancer. As a factor related to cell death, FSP1, a lipid peroxidation regulator, was originally called apoptosis-inducing factor 2. However, with increased research on ferroptosis, its function as a negative regulator of ferroptosis was recognized (14, 15). Thus, GCH1 may possess undiscovered functions related to tumor survival and progression. In addition, although this study did not find an inverse correlation with lipid peroxidation markers, if we assume that low expression of GCH1 potentially makes the cell more susceptible to oxidative stress caused by lipid peroxidation, it is possible that DNA damage caused by oxidative stress, including lipid peroxidation, may also have an effect. This may cause additional genetic abnormalities and contribute to the acquisition of drug resistance to chemotherapy (35, 36). Furthermore, it is possible that oxidative stress alters epigenetic mechanisms and changes the regulatory elements of gene expression, thereby promoting cancer progression and resistance to chemotherapy (37).

In the present study, no significant inverse correlation was observed between 4-HNE accumulation and the expression of lipid peroxidation regulators in the esophageal cancer cells; however, a positive correlation was observed. Various reports have been published on the relationship between oxidative stress markers and antioxidant enzyme expression. A negative correlation between relative GPX4 overexpression and lipid peroxidation markers has been observed in diffuse large B-cell lymphoma, lung squamous cell carcinoma, and colorectal cancer (18, 20, 23). In contrast, a positive correlation has been found between 4-HNE accumulation and FSP1 expression in diffuse large B-cell lymphoma (19). In hepatocellular carcinoma, no significant inverse correlation has been found between the accumulation of 4-HNE and lipid peroxidation regulators (21).

4-HNE is metabolized by a variety of enzymes such as aldo-keto reductase, aldehyde dehydrogenase, and glutathione S-transferase (38–40). Decreased 4-HNE accumulation is a poor prognostic factor in hepatocellular carcinoma, and intracellular 4-HNE metabolism is controlled by SMARCA4. The control of 4-HNE metabolism by SMARCA4 is important in promoting the survival of hepatocellular carcinoma cells (21). Although no significant correlation was observed between 4-HNE expression and the prognosis in esophageal cancer tissues, 4-HNE metabolism by various enzymes, in addition to the extent of 4-HNE accumulation as lipid peroxidation is regulated by GPX4 and FSP1, may be observed and should be evaluated together with other lipid peroxidation markers.

Anticancer therapy for esophageal cancer is centered on apoptosis-inducing agents, such as cisplatin (8, 10). However, resistance to cisplatin is considered a major challenge in many cases (7). To overcome this, ferroptosis, a nonapoptotic cell death process, has attracted considerable attention (11). In a study of ferroptosis-induced cell death in lung squamous cell carcinoma, a dramatic induction of ferroptosis was reported in lung squamous cell carcinoma cells exposed to the GPX4 inhibitor RSL3, an inhibitor of FSP1, and iFSP1 (18). Moreover, simultaneous regulation of GPX4 and FSP1 reportedly induces ferroptosis in esophageal cancer cell lines (24). In our *in vitro* experiments, simultaneous inhibition of GPX4 and FSP1 induced only a mild effect on cell death, whereas simultaneous inhibition of GCH1 dramatically enhanced ferroptosis induction. The results of this study have two major implications: First, GPX4 and FSP1 inhibition alone was not sufficient to induce ferroptosis in all the cancer cells. Second, GCH1 inhibition may induce effective ferroptosis by showing a synergistic interaction with simultaneous GPX4 and FSP1 inhibition, even in cells that are resistant to cell death. The mechanism is based on the complementary roles of GPX4, FSP1, and GCH1 in the regulation of ferroptosis; GPX4 and FSP1 are known inhibitors of ferroptosis, and their simultaneous inhibition interferes with the ability to prevent lipid peroxidation in cells. GCH1 is involved in the synthesis of tetrahydrobiopterin (BH4) and is thought to be involved in the antioxidant defense of cells. By knocking down GCH1, it is thought that the ability of cells to resist oxidative stress was further impaired, and the ferroptosis response when GPX4 and FSP1 were inhibited was enhanced. Many reports support that the simultaneous inhibition of GPX4 and FSP1 can induce favorable ferroptosis (14, 15, 41, 42). However, mutations in TP53, the most frequent mutations in ESCC, have been shown to affect ferroptosis (43). Although the effects of TP53 on ferroptosis are controversial, mutant TP53 APR-246 has been reported to induce ferroptosis more efficiently in hematopoietic malignancies, supporting the negative regulation of ferroptosis by mutant TP53 (44). According to a database search (https://pickles.hart-lab.org/), the KYSE-150 cells used in this study harbored a hotspot mutation in TP53, which may not induce ferroptosis when GPX4 and FSP1 are inhibited simultaneously. TP53 is a genetic mutation that occurs not only in ESCC but also in various cancers and may cause resistance to cisplatin and ferroptosis. Therefore, targeting GCH1, in addition to depleting GPX4 and FSP1, can effectively induce ferroptosis in cancer cells that are resistant to apoptosis. Furthermore, the development of a new therapeutic approach targeting GCH1 is expected to address the resistance to ferroptosis in cancer cells resulting from *TP53* mutations and other factors when GPX4 and FSP1 are targeted.

Recently, immune checkpoint inhibitors have gained attention as promising therapeutic approaches for cancer therapy (45). Cancer cells create an immunosuppressive environment by utilizing ligands and receptors (46). The programmed cell death protein-1/programmed death-ligand 1 and programmed cell death protein-1/programmed death-ligand 2 pathways and upregulation of anti-cytotoxic T lymphocyte-associated antigen 4 on the T cell surface are used to evade host immune responses (47). Recent studies have shown that GCH1 is involved in immunity and is a competent regulator of ferroptosis. Specifically, inhibition of BH4 synthesis *in vivo* reportedly abolishes T cell-mediated autoimmunity and allergic inflammation, whereas GCH1 overexpression increases BH4 levels, leading to increased responses by CD4- and CD8-expressing T cells and enhanced antitumor activity *in vivo* (48). Thus, GCH1 is not only a target for cancer therapy focusing on ferroptosis but also a potential strategy for new cancer therapies that utilize the immune response function of GCH1. Utilizing such synergistic interactions is important for developing effective treatment strategies, especially for cancers such as ESCC that are generally resistant to conventional treatments. The synergistic effects observed in our study indicate that targeting multiple pathways simultaneously may overcome resistance and induce cell death more effectively.

This study had some limitations. The expression levels of GPX4, FSP1, and GCH1 in ESCC vary, and we were unable to explore the molecular mechanisms regulating their expression in this study. In particular, we were unable to quantitatively analyze BH4, a synthetic target of GCH1. Therefore, the interpretation of GCH1-mediated anti-lipid peroxidation effects must also consider the BH4 concentration. Recently, it was reported that dihydroorotate dehydrogenase and aldo-keto reductase, in addition to GPX4, FSP1, and GCH1, are involved in the regulation of ferroptosis; therefore, it is desirable to examine the expression of these factors and analyze their molecular mechanisms in esophageal cancer cells (49, 50). Although this study confirmed the effect of ferroptosis *in vitro*, *in vivo* studies are warranted in the future to validate the findings. Regarding the design of the clinicopathological study, the present study was retrospective; thus, prospective multicenter studies are desired.

Collectively, these results suggest that GCH1 is an important prognostic factor in ESCC and may provide a basis for new therapeutic strategies to control lipid peroxidation. Future studies will improve our understanding of the role and function of GCH1 and encourage the development of new approaches for ESCC treatment.

## Data Availability

The datasets presented in this study can be found in online repositories. The names of the repository/repositories and accession number(s) can be found in the article/**Supplementary Material**.
