# Supplementary material for: Prognostic significance and therapeutic potential of guanosine triphosphate cyclohydrolase 1 in esophageal squamous cell carcinoma: clinical implications of ferroptosis and lipid peroxidation regulation
Source: Front Oncol. 2024 Dec 11;14:1459940. doi: 10.3389/fonc.2024.1459940 (PMC11668648; doi:10.3389/fonc.2024.1459940)
Supplement: Supplementary file 1 [file DataSheet1.docx]

Supplementary Material

# Supplementary Tables and Figures

| Supplementary Table 1. Correlation between 4-HNE(C) accumulation and clinicopathological features | | | | |  |
| --- | --- | --- | --- | --- | --- |
|  |  | **4-HNE(C)** | |  |  |
| **Variable** |  | **Low** | **High** | ***p*-value** |  |
| **Age** | <69 y | 64 | 89 | 0.614 |  |
|  | ≥69 y | 71 | 88 |  |  |
| **Sex** | Male | 115 | 146 | 0.522 |  |
|  | Female | 20 | 31 |  |  |
| **Smoking** | None | 35 | 40 | 0.495 |  |
|  | Exist | 100 | 137 |  |  |
| **Alcohol** | None | 23 | 27 | 0.670 |  |
|  | Exist | 112 | 150 |  |  |
| **Differentiation** | Well/moderate | 83 | 121 | 0.250 |  |
|  | Poor/unknown | 52 | 56 |  |  |
| **Lymph node metastases** | None | 34 | 59 | 0.119 |  |
|  | Exist | 101 | 118 |  |  |
| **Lymphovascular invasion** | None | 44 | 69 | 0.244 |  |
|  | Exist | 91 | 108 |  |  |
| **Vessel invasion** | None | 17 | 41 | **0.017** |  |
|  | Exist | 118 | 136 |  |  |
| **Pathological tumor stage** | I–II | 47 | 81 | 0.051 |  |
|  | III–IV | 88 | 96 |  |  |
|  |  |  |  |  |  |

4-HNE: 4-hydroxy-2-nonenal, (C): cytoplasmic expression

|  |  |  |  |  |  |
| --- | --- | --- | --- | --- | --- |
| **Supplementary Table 2. Correlation between 4-HNE(N) accumulation and clinicopathological features** | | | | |  |
|  |  | **4-HNE(N)** | |  |  |
| **Variable** |  | **Low** | **High** | ***p*-value** |  |
| **Age** | <69 y | 68 | 85 | 0.113 |  |
|  | ≥69 y | 85 | 74 |  |  |
| **Sex** | Male | 131 | 130 | 0.356 |  |
|  | Female | 22 | 29 |  |  |
| **Smoking** | None | 36 | 39 | 0.836 |  |
|  | Exist | 117 | 120 |  |  |
| **Alcohol** | None | 23 | 27 | 0.639 |  |
|  | Exist | 130 | 132 |  |  |
| **Differentiation** | Well/moderate | 106 | 98 | 0.115 |  |
|  | Poor/unknown | 47 | 61 |  |  |
| **Lymph node metastases** | None | 37 | 56 | **0.033** |  |
|  | Exist | 116 | 103 |  |  |
| **Lymphovascular invasion** | None | 54 | 59 | 0.739 |  |
|  | Exist | 99 | 100 |  |  |
| **Vessel invasion** | None | 16 | 42 | **<0.001** |  |
|  | Exist | 137 | 117 |  |  |
| **Pathological tumor stage** | I–II | 53 | 75 | **0.024** |  |
|  | III–IV | 100 | 84 |  |  |
|  |  |  |  |  |  |

4-HNE: 4-hydroxy-2-nonenal, (N): nuclear expression

| **Supplementary Table 3. Correlation between 4-HNE(C) and 4-HNE(N) accumulation and clinicopathological features** | | | | |  |  |
| --- | --- | --- | --- | --- | --- | --- |
|  |  | **4-HNE(C) and 4-HNE(N)** | |  |  |  |
| **Variable** |  | **Others** | **High** | ***p*-value** |  |  |
| **Age** | <69 y | 88 | 65 | 0.074 |  |  |
|  | ≥69 y | 107 | 52 |  |  |  |
| **Sex** | Male | 164 | 97 | 0.782 |  |  |
|  | Female | 31 | 20 |  |  |  |
| **Smoking** | None | 46 | 29 | 0.810 |  |  |
|  | Exist | 149 | 88 |  |  |  |
| **Alcohol** | None | 30 | 20 | 0.690 |  |  |
|  | Exist | 165 | 97 |  |  |  |
| **Differentiation** | Well/moderate | 130 | 74 | 0.538 |  |  |
|  | Poor/unknown | 65 | 43 |  |  |  |
| **Lymph node metastases** | None | 50 | 43 | **0.037** |  |  |
|  | Exist | 145 | 74 |  |  |  |
| **Lymphovascular invasion** | None | 67 | 46 | 0.377 |  |  |
|  | Exist | 128 | 71 |  |  |  |
| **Vessel invasion** | None | 24 | 34 | **<0.001** |  |  |
|  | Exist | 171 | 83 |  |  |  |
| **Pathological tumor stage** | I–II | 71 | 57 | **0.032** |  |  |
|  | III–IV | 124 | 60 |  |  |  |
|  |  |  |  |  |  |  |

4-HNE: 4-hydroxy-2-nonenal, (C): cytoplasmic expression, (N): nuclear expression

| **Supplementary Table 4. Correlation between GPX4 accumulation and clinicopathological features** | | | | |  |
| --- | --- | --- | --- | --- | --- |
|  |  | **GPX4** | |  |  |
| **Variable** |  | **Low** | **High** | ***p*-value** |  |
| **Age** | <69 y | 67 | 86 | 0.205 |  |
|  | ≥69 y | 81 | 78 |  |  |
| **Sex** | Male | 124 | 137 | 0.953 |  |
|  | Female | 24 | 27 |  |  |
| **Smoking** | None | 35 | 40 | 0.878 |  |
|  | Exist | 113 | 124 |  |  |
| **Alcohol** | None | 25 | 25 | 0.691 |  |
|  | Exist | 123 | 139 |  |  |
| **Differentiation** | Well/moderate | 110 | 94 | **0.001** |  |
|  | Poor/unknown | 38 | 70 |  |  |
| **Lymph node metastases** | None | 46 | 47 | 0.640 |  |
|  | Exist | 102 | 117 |  |  |
| **Lymphovascular invasion** | None | 54 | 59 | 0.925 |  |
|  | Exist | 94 | 105 |  |  |
| **Vessel invasion** | None | 24 | 34 | 0.305 |  |
|  | Exist | 124 | 130 |  |  |
| **Pathological tumor stage** | I–II | 59 | 69 | 0.692 |  |
|  | III–IV | 89 | 95 |  |  |
|  |  |  |  |  |  |

GPX4: glutathione peroxidase 4

| **Supplementary Table 5. Correlation between FSP1 accumulation and clinicopathological features** | | | | |  |
| --- | --- | --- | --- | --- | --- |
|  |  | **FSP1** | |  |  |
| **Variable** |  | **Low** | **High** | ***p*-value** |  |
| **Age** | <69 y | 61 | 92 | **0.038** |  |
|  | ≥69 y | 82 | 77 |  |  |
| **Gender** | Male | 119 | 142 | 0.847 |  |
|  | Female | 24 | 27 |  |  |
| **Smoking** | None | 37 | 38 | 0.485 |  |
|  | Exist | 106 | 131 |  |  |
| **Alcohol** | None | 26 | 24 | 0.339 |  |
|  | Exist | 117 | 145 |  |  |
| **Differentiation** | Well/moderate | 100 | 104 | 0.120 |  |
|  | Poor/unknown | 43 | 65 |  |  |
| **Lymph node metastases** | None | 39 | 54 | 0.367 |  |
|  | Exist | 104 | 115 |  |  |
| **Lymphovascular invasion** | None | 58 | 55 | 0.142 |  |
|  | Exist | 85 | 114 |  |  |
| **Vessel invasion** | None | 17 | 41 | **0.005** |  |
|  | Exist | 126 | 128 |  |  |
| **Pathological tumor stage** | I–II | 54 | 74 | 0.281 |  |
|  | III–IV | 89 | 95 |  |  |
|  |  |  |  |  |  |

FSP1: ferroptosis suppressor protein

**Supplementary Figure 1.** Suppression of guanosine triphosphate cyclohydrolase 1 (GCH1) enhances nonapoptotic cell death by inhibiting glutathione peroxidase 4 (GPX4) and ferroptosis suppressor protein 1 (FSP1). Detailed cell death ratios and standard deviations for each experiment are given in this figure.
