# Supplementary figures and images for: Prognostic significance and therapeutic potential of guanosine triphosphate cyclohydrolase 1 in esophageal squamous cell carcinoma: clinical implications of ferroptosis and lipid peroxidation regulation
Source: Front Oncol. 2024 Dec 11;14:1459940. doi: 10.3389/fonc.2024.1459940 (PMC11668648; doi:10.3389/fonc.2024.1459940)

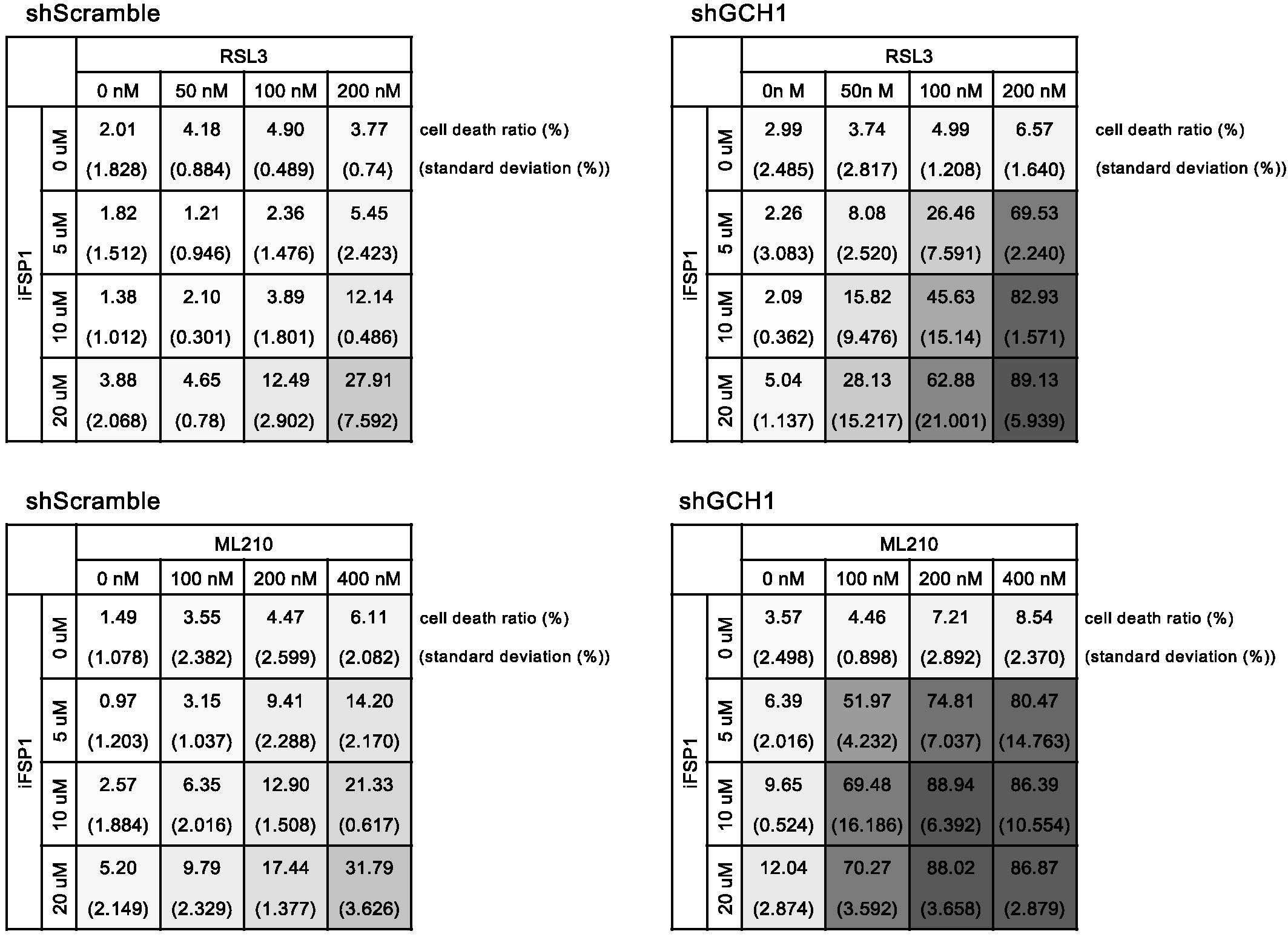

Supplement: Supplementary file 2 [file Image1.tif]
